# A cross‐sectional survey of mental health service users’, carers’ and professionals’ priorities for patient safety in the United Kingdom

**DOI:** 10.1111/hex.12805

**Published:** 2018-08-17

**Authors:** Kathryn Berzins, John Baker, Mark Brown, Rebecca Lawton

**Affiliations:** ^1^ School of Healthcare University of Leeds Leeds UK; ^2^ Social Spider CIC London UK; ^3^ School of Psychology University of Leeds Leeds UK

**Keywords:** attitude of health personnel, caregivers, health services research, inpatients, mental health services, patient safety, surveys and questionnaires

## Abstract

**Background:**

Establishing patient safety priorities in psychiatry has received less international attention than in other areas of health care. This study aimed to identify safety issues as described by people in the United Kingdom identifying as mental health service users, carers and professionals.

**Methods:**

A cross‐sectional online survey was distributed via social media. Identified safety issues were mapped onto the Yorkshire Contributory Factors Framework (YCFF) which categorizes factors that contribute to patient safety incidents in general hospital settings. Service user and carer responses were described separately from professional responses using descriptive statistics.

**Results:**

One hundred and eighty‐five responses from 95 service users and carers and 90 professionals were analysed. Seventy different safety issues were identified. These were mapped onto the 17 existing categories of the YCFF and two additional categories created to form the YCFF‐MH. Most frequently identified issues were as follows: “Individual characteristics” (of staff) which included competence and listening skills; “Service process” that contained concerns about waiting times; “Management of staff and staffing levels” dominated by staffing levels; and “External policy context” which included the overall resourcing of services. Professionals identified staffing levels and inadequate community provision more frequently than service users and carers, who in turn identified crisis care more frequently.

**Conclusions:**

This study updates knowledge on stakeholder perceived safety issues across mental health care. It shows a far broader range of issues relating to safety than has previously been described. The YCFF was successfully modified to describe these issues and areas for further coproduced research are suggested.

## 1 INTRODUCTION

### 1.1 Patient safety in health care

Improving patient safety has been a priority in health care for nearly two decades^1^ and the subject of a significant body of research and innovation. Patient safety in health care is a broad area, although typical interventions would be aimed at reducing medication errors, preventing hospital‐acquired infections, reducing falls or pressure sores and improving incident reporting.^2^ There has been a significant amount of research carried out to this end supporting numerous improvements to practice.^3^ In the pursuit of safety, the focus is on identifying negative outcomes and reducing the likelihood of their future occurrence; the publication of the Draft Health Service Safety Investigations Bill seeks to create a legal structure for this in England. To this end, researchers have sought to establish, based on the evidence, the factors that contribute to patient safety incidents. The Yorkshire Contributory Factors Framework (YCFF)^4^ was developed by reviewing 83 empirical studies of the factors causing different types of patient safety incidents, although only two included patient perspectives. It provides a unique and validated framework to describe factors that contribute to safety incidents in general hospital settings (although it has subsequently been adapted for use in primary care^5^) ranging from proximal, for example individual staff factors; to distal, for example organisational policy. One aim of the framework is to encourage risk managers and incident investigators to move beyond the proximal causes (e.g. violation of a rule or inexperience of the nurse) to a consideration of the local working conditions and the organisational culture in which patient safety incidents are more likely to occur.

Contrary to developments in general hospital care, there has been little parallel research into the identification of safety issues in mental health care services. Research into patient safety in mental health care has been dominated by the process of individual risk assessment with a focus on the prevention of suicide and homicide,^6^ an approach that can be incompatible with recovery‐orientated mental health care.^7^ Recent evidence has shown this individual risk assessment frequently does not involve the service user and their families and thus does not take into account their priorities.^8^ There have been three reviews of broader safety issues in mental health care; all identified similar issues including violence and aggression, suicide and self‐harm, seclusion and restraint, substance misuse and staff retention.^9^, ^10^, ^11^ All recommended further research to better identify and understand the issues so that effective interventions can be developed. A recent Delphi study primarily of professionals reported priorities for further research about safety in mental health care, and these included how service users might contribute to their own safety particularly if they self‐harm, individual safety planning and the reduction in restrictive practices.^12^

In the past decade, UK mental health services have continued to change at a rapid pace; detentions under the Mental Health Act have increased,^13^ inpatient beds reduced,^14^ staff numbers have fallen,^15^ yet perceptions of safety issues from the perspectives of service users and carers are largely unexplored. There has been little attention paid to this area in the United Kingdom, despite high‐profile failures in service provision,^16^ for example a mental health trust was the first to be prosecuted by the Care Quality Commission(CQC) after the Francis Report, (a public inquiry into care failures at Mid Staffordshire Foundation Trust^17^) for failing to provide safe care and treatment.^18^ In the first quarter of 2016, there were 223 276 patient safety incidents in mental health services,^19^ 13% of all incidents recorded by the UK National Health Service(NHS). Furthermore, the CQC has reported that 40% of NHS core mental health services are either inadequate or require improvement in relation to safety. Key concerns were about physical environments, staffing, coercive practices and access to services including crisis care.^20^

This study aimed to identify current safety issues in UK mental health care as described by service users, carers and professionals.

## 2 METHODS

This was a cross‐sectional semi‐structured survey accessed by a web address distributed across the United Kingdom via social media (twitter). Twitter was selected as the primary method for distribution due to its ability for social reach into specific communities of interest, in this case mental health professionals, service users and carers. Using social media has been shown to be a cost‐effective and rapid way in which to recruit people into research, particular those from potentially stigmatised groups, and the peer network structures of platforms such as twitter mean that users can recruit other users.^21^ An invitation to take part in the survey was tweeted by the authors and retweeted in turn by their networks. No attempts were made to track the tweets, although anonymised traffic to the survey was monitored. Participants were eligible for inclusion if they were aged over 18, had recent experience (within the past two years) of using, caring for someone using, or working in mental health services.

### 2.1 Data collection

The electronic survey was created by the authors using the Bristol Online Survey platform which collates responses into a database and records IP addresses to prevent multiple responses from any one internet connection. The study was open between September and December 2016. The survey consisted of 18 questions specifically designed for this study. The questions about safety issues were consciously open and exploratory due to the broad range of potential safety issues identified both in the literature and during initial consultations with stakeholders. Participants were routed in one of three different ways depending on whether they primarily identified as a service user, carer or professional. The survey asked about demographic characteristics (age, gender, location), and asked for free‐text responses to the question: “Please tell me what you think are the things that affect safety in mental health care?” The full survey is published at https://doi.org/10.6084/m9.figshare.6300800.v1.

Approval for the study was granted by the University of Leeds, School of Healthcare Research Ethics Committee (ref. HREC15‐059). Information was provided about both the survey and sources of support at the beginning and consent was implied by completion and submission of the survey.

### 2.2 Data analysis

All the individual safety issues identified by participants were read and then coded by two researchers (KB and JB). Initial codes were generated, for example: “poor crisis support” or “alcohol on wards” resulting in 70 codes that accounted for all responses. These codes were then mapped onto the Yorkshire Contributory Factors Framework (YCFF) to aid interpretation. The decision to use the YCCF was made as it is theoretically based, there was no similar framework specific to mental health care available and it was not possible to develop one in the same way as there is not the primary research to draw upon. The framework consists of latent external factors (government policy), organisational factors (scheduling and bed management), local working conditions (staff workload), situational factors (individual staff) and active failures (mistakes), along with two cross‐cutting themes of communication and safety culture. The mapping of codes onto the YCCF was initially carried out by KB and JB and discrepancies discussed with RL (the original author of the YCCF), before amendments were made (described below) and consensus gained. The free‐text responses were coded using the modified YCCF (supported by Microsoft Excel) to provide illustration of individual issues.

The survey findings were numerically coded for analysis using SPSS 22,^22^ and descriptive statistics were used to describe the sample characteristics. Comparisons of service user and carer and professionals groups were conducted using chi‐square tests; further comparisons were carried out between those service users who had recently experienced inpatient treatment and those who had not, and between those staff whose recent employment was in inpatient services or community services.

### 2.3 Patient involvement in the design and conduct of the study

Mental health service user and carer representatives and other stakeholders (e.g. collective advocacy organisations, mental health professionals and policymakers) were involved throughout this study. The research aims arose from social media discussions with a range of people, including (ex)service users, family members, carers and professionals in a range of roles about improving and understanding key safety issues in mental health services. The subsequent survey was developed following discussions with (ex)service users about patient safety in mental health settings. A number of active social media campaigners, including (ex)service users, were contacted about the specific design and content of the survey. They provided feedback on the questions and participant information resulting in adaptations being made to the wording of the survey, and this continued when the survey was launched after feedback from respondents, for example the additional of an employment category. These individuals assisted with maximising response rates using their substantial networks to actively promote the distribution of the survey. Author MB is mental health design researcher, writer and consultant. MB has direct lived experience of mental health difficulty and 12 years of experience in developing and delivering mental health projects with a strong participatory element. He has been involved for the duration of the study having been part of the initial application for funding, codesigned the survey, led the distribution through social media and subsequently provided input into the analysis and reporting of this study.

## 3 FINDINGS

The survey received 188 responses from across the United Kingdom although three were excluded from the analysis as they were less than 50% complete. The analysed sample of 185 consisted of 90 professionals (48.6%), 77 service users (41.6%) and 18 carers (9.7%). For the purpose of analysis service, users’ and carers’ views were combined and presented separately from professionals. The demographic characteristics of the respondents are shown in Table 1.

**Table 1:** Sample characteristics

	Service users and carers		Professionals	
	%	n	%	n
Age				
18‐25	15	14	1	1
26‐35	20	19	18	16
36‐45	19	18	31	28
46‐55	32	30	37	33
56‐65	13	12	13	12
>65	1	1	0	0
Gender				
Male	23	22	29	26
Female	75	71	71	64
Ethnicity				
White British	76	72	72	65
British Asian	2	2	4	4
White Other	7	7	13	12
Other	4	4	2	2
UK region				
North West	11	10	18	16
N. Yorks & Humber	12	11	20	18
South West	7	7	8	7
South East	16	15	9	8
London	16	15	14	13
East of England	6	6	3	3
East Midlands	8	8	9	8
West Midlands	8	8	4	4
Wales	4	4	3	3
Scotland	8	8	7	6
North East	2	2	4	4
Contact with services				
2‐5 y	33	31	16	14
6‐10	27	26	14	13
11‐15	19	18	19	17
16‐20	4	4	16	14
>20	14	13	35	32
Type of service contact in past 2 y				
CMHT inpatient services	47	45	23	21
GP	17	16	31	28
Voluntary	25	24	6	5
Organisation	6	6	7	6
Other	3	3	29	26

There were more women than men in both groups and the majority of respondents described themselves as White British. The largest percentage of service users and carers (33%) had been in contact with services for five years or less with most of their contact over the previous two years with Community Mental Health Teams (CMHT) (48%), although 40% had experienced compulsory treatment in the past. Registered nurses were the largest professional group (n = 20; 22%), 17 respondents described themselves as managerial (18%) rather than by professional orientation. Service users and carers were asked to describe their mental health problem in their own words; many provided diagnoses but others used non‐diagnostic terms such as “severe and enduring” (21%). Nearly half said they experienced depression and anxiety (n = 38; 44%); 17% PTSD, 16% personality disorder, 10% schizophrenia and psychosis and 9% bipolar disorder.

### 3.1 Safety issues identified in mental health care

**Figure 1:**
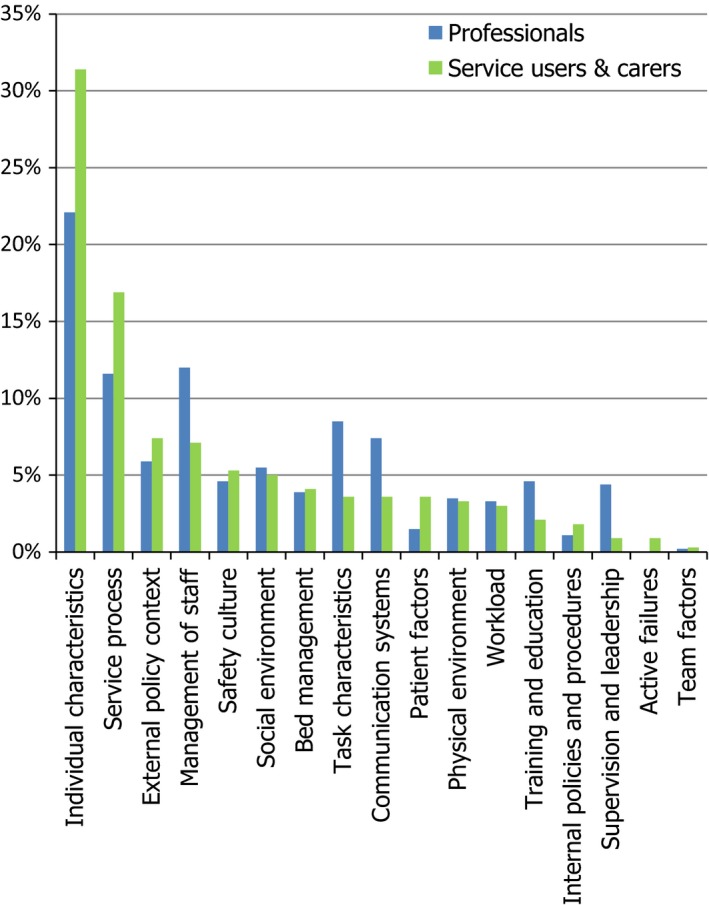
Summary of responses by YCCF‐MH category

There were 796 individual responses identifying safety issues which after coding resulted in 70 first‐level categories. The majority of these codes fitted well within the YCCF but 20% did not. Following scrutiny of these codes by the author team, a decision was made to add two new categories. These were termed “Social environment” which took into account concerns about the social aspects of the service environment, for example other patients’ behaviour on mental health wards; and “Service process” which took into account both gaining access to and discharge from services, for example, not being able to access crisis care or being discharged from hospital before feeling suitably recovered. These were factors that the original YCCF did not address as it was derived exclusively from general hospital studies^4^ and this survey asked about experiences across all mental health services in both hospital and community because there can be regular transitions between both. The 70 categories were subsequently mapped onto the amended YCCF‐Mental Health (MH) (frequencies of category responses shown in Figure 1). Table 2 shows the full list of safety issues under all the YCCF‐MH headings and Table 3 the most frequently cited safety issues.

**Table 2:** List of 70 safety issues under YCCF‐MH headings

YCCF‐MH and subcategories	Total		Service users and carers		Professionals	
	N of cases	% of cases	N of cases	% of cases	N of cases	% of cases
Individual characteristics^a^	111	60	52	58	59	62
Staff incompetence	36	20	18	20	18	19
Poor staff attitudes	23	13	10	11	13	14
Staff not listening	20	11	5	6	15	16
Staff not understanding	20	11	7	8	13	14
Staff lack of compassion	17	9	7	8	10	11
Staff burnout	16	9	11	12	5	5
Low quality of care	15	8	6	7	9	10
Lack of therapeutic relationships	12	7	9	10	3	3
Stigma from staff	11	6	5	6	6	7
Staff inexperience	9	5	8	9	1	1
Untrustworthy staff	8	4	3	3	5	5
Staff morale	6	3	3	3	3	3
Staff disbelieving	2	1	1	1	1	1
Staff having poor English	2	1	1	1	1	1
Service process^a^ (New category for YCCF‐MH)	70	38	32	36	38	40
Waiting times	33	18	12	13	21	23
High threshold for support	19	10	11	12	8	9
Poor community provision	15	8	10	11	5	5
Poor crisis support	11	6	1	1	10	11
Poor continuity of care	10	6	7	8	3	3
Poor access to psychological therapies	9	5	5	6	4	4
No early intervention provision	6	3	2	2	4	4
Premature discharge from hospital	6	3	5	6	1	1
Poor transition from CAMHS	1	1	0	0	1	1
Management of staff and staffing levels^a^	69	37	47	52	22	23
Low staffing levels	65	36	47	52	18	19
High use of agency/bank staff	7	4	4	4	3	3
High number of staff vacancies	4	2	3	3	1	1
No visible staff on wards	3	2	1	1	2	2
External policy context^a^	49	27	24	29	23	24
Underfunding of mental health care	36	20	15	17	21	23
Societal stigma	10	6	7	8	3	3
Wider social problems	7	4	5	6	2	2
NHS targets	4	2	3	3	1	1
NHS policies	3	2	3	3	0	0
Legal issues	2	1	1	1	1	1
Task characteristics^a^	40	22	30	33	10	11
Poor risk assessment	31	17	23	26	8	9
Poor care planning	17	9	13	14	4	4
Staff only able to “firefight”	3	2	3	3	0	0
Communication systems^a^	34	18	23	26	11	12
Poor communication	28	15	21	23	7	8
Inaccurate information	5	3	2	2	3	3
Ineffective use of technology	5	3	5	6	0	0
Administration burden	5	3	5	6	0	0
Confidentiality	2	1	0		2	2
Poor feedback mechanisms	1	1	1	1	0	0
Social environment^a^ (New category for YCCF‐MH)	34	18	20	22	14	15
Substance misuse	11	6	9	10	2	2
Patient acuity	11	6	8	9	3	3
Threats from other patients	10	6	1	1	9	10
Other patients boredom	5	3	3	3	2	2
Other patients self‐harming	3	2	2	2	1	1
Violence from other patients	2	1	2	2	0	0
Safety culture^a^	29	16	15	17	14	15
Service culture	18	10	14	16	4	4
Coercion by staff	13	7	4	4	9	10
Lack of coproduction	4	2	2	2	2	2
Poor complaints and whistleblowing procedures	3	2	1	1	2	2
Disregard for psychological safety	1	1	0	0	1	1
Bed management^a^	30	16	16	18	14	15
Lack of inpatient beds	26	14	14	16	12	13
Use of out of area treatment	5	3	4	4	1	1
Placing children on adult wards	1	1	0	0	1	1
Physical environment^a^	23	12	15	17	8	8
Unsafe environment	22	12	15	17	7	8
Access to ligature points	5	3	1	1	4	4
Staff workload^a^	22	12	14	16	8	8
Inadequate time with staff	16	9	12	13	4	4
Adequate monitoring on wards	7	4	3	3	4	4
Caseload size	2	1	0	0	2	2
Training^a^	26	14	19	21	7	7
Inadequate staff training	24	13	18	20	6	7
Poor staff physical health training	4	2	3	3	1	1
Supervision and leadership^a^	20	11	17	19	3	3
Poor supervision	14	8	14	16	0	0
Poor leadership	9	5	6	7	3	3
Patient factors^a^	17	9	6	7	11	12
Medication issues	16	9	6	7	10	11
Increased MHA detentions	3	2	1	1	2	2
Policies and procedures^a^	11	6	5	6	6	6
Poor carer support/involvement	7	4	3	3	4	4
Poor legal advice/advocacy	4	2	2	2	2	2
Active failures^a^	3	2	0	0	3	3
Abuse from staff	3	2	0	0	3	3
Team factors^a^	2	1	1	1	1	1
Poor teamwork	2	1	1	1	1	1

a Total number of cases referring to factor at least once.

**Table 3:** Frequently identified safety issues

YCCF	Total		Service users & carers		Professionals	
	N	% of cases)	N	% of cases)	N	% of cases)
Individual factors^a^	111	60	59	62	52	58
Staff competence	36	19	18	20	18	19
Staff attitudes	23	13	13	14	10	11
Staff not listening*	20	10	15	16	5	6
Staff not understanding	20	10	13	14	7	8
Staff lack of compassion	17	9	10	11	7	8
Staff burnout	16	9	5	5	11	12
Service process^a^	70	38	38	40	32	36
Waiting times	33	18	21	23	12	13
High threshold for support	19	10	8	9	11	12
Poor community provision	15	8	5	5	10	11
Poor crisis support**	11	6	10	11	1	0
Poor continuity of care	10	6	3	3	7	8
Management of staff & staffing levels^a^	69	37	22	23	47	52
Poor staffing levels***	65	36	18	19	47	52
External policy context^a^	49	27	23	24	26	29
Underfunding of mental health care	36	20	21	23	15	17

a Total number of cases referring to factor at least once.

*95 CI 0.2404‐19.7624; χ^2^ = 4.645; *P* = .0311; **95 CI 2.6706‐18.1389; χ^2^ = 7.982; *P* = .0047; ***95 CI 18.7287‐45.9289; χ^2^ = 21.988; *P* < .0001.

“Individual (staff) factors” was the most frequently cited YCCF‐MH category of both service user and carers and professionals. The second YCCF‐MH categories differed between groups with service users describing “Service process” followed by “External policy context.” Professionals cited “Management of staff and staffing levels” as the second with “Service process” coming third.

Within the “Individual (staff) factors” category, both groups most frequently referred to staff competence followed by poor attitudes. Staff competence was often illustrated by respondents describing specific issues such as staff not being able to respond to service user distress:

Lack of skills, confidence, and knowledge of staff to deal with challenging behaviour and risk. Professional #159

Significantly more service users than professionals thought staff not listening was a safety issue (*P* < .05, see Table 1), some respondents described their views being dismissed:

Truly listening to service user/carers views rather than [saying] ‘well that's your perspective’. Service user #36

There were differences between the safety issues identified by staff depending on whether they worked in community or inpatient services. Significantly fewer inpatient staff gave responses related to individual staff characteristics as a safety issue (Difference 25; 95 CI 27.8169 to 65.4693; χ^2^ = 19.250; *df* 1; *P* < .0001).

The issue of burnout was more frequently identified by professionals; this was seen as undermining their ability to provide safe care:

Staff should be supported with adequate supervision, training and manageable case loads so that they do not experience burnout which can impact on patient safety. Professional #49

The new YCCF‐MH category of “Service process” had agreement between the two groups about waiting times and high thresholds for accessing support as being the main threats to service user safety within this category. Some service users reported being told their needs were not severe enough to receive a service and others of having to wait longer for more intensive support:

[I] was also told if my risk/need was lower I'd wait [a] much shorter [time] as more staff [would be] available. Service user #77

Significantly more service users were concerned about difficulties in accessing specific crisis support when in the community (*P* < .005, see Table 1), although professionals also identified lack of community services in general as a threat to safety, particularly for people moving back to the community from inpatient care:

Lack of resources result in service users being discharged from inpatient settings to community services that are unable to manage risk safely and provide continuity of care. Professional #116

A larger proportion of service users and carers with recent experience of community‐based services had identified issues relating to service process as a safety issue (Difference 35; 95 CI 4.4066 to 58.6881; χ^2^ = 6.476; *df* 1; *P* < .05).

The category of “Management of staff and staffing levels” was dominated by staffing numbers; the second commonest safety concern for professionals, identified significantly more frequently than by service users (*P* < .005, see Table 1):

Due to shortage of staff inpatients are not observed or interacted with… community patients are not seen enough resulting in care being delivered in crisis situation rather than planned work. Patients are being placed miles from home so staff have no previous knowledge and people are placed in inadequate community placements due to lack of alternatives. Professional #43

The service users and carers with recent experience of inpatient services were more likely to have identified short staffing as a safety issue (Difference 19; 95 CI‐1.8193 to 47.8663; χ^2^ = 4.544; *df* 1; *P* < .05). Correspondingly, a greater proportion of professionals currently employed in inpatient services also reported staffing levels to be a safety issue when compared with those working in the community (Difference 38; 95 CI 15.2331 to 54.7176; χ^2^ = 11.265; *df* 1; *P* < .05).

The category “External policy context” was dominated by concern about the safety implications of overall resourcing of mental health services. Both groups most frequently described this in terms of government cuts affecting mental health funding at local levels:

Inadequate services (as a result of poor funding and pressures caused by other service cuts) Professional #101

Overall, the defining feature of the majority of the safety issues raised by participants was their reference to staff characteristics:

…the risks are more caused by people/human error (quality of staffing and management) than broken equipment. Service user #124

## 4 DISCUSSION

This paper reports mental health service users’, carers’ and professionals’ views of current safety issues in UK mental health services, across both inpatient and community settings. The safety focus in mental health has been confined to risk, homicide, suicide and deaths. Broader research which considers systemic safety issues does not appear to have been as prolific as in other areas of health care in the last ten years.^9^, ^10^, ^11^ During this decade, there have been continuing constraints on services as a result of austerity measures^13^, ^14^, ^15^ and the issue of safety has dramatically increased in prominence after inquiries into care failures in the NHS in both general medicine and mental health care.^17^, ^18^

The data we present update the issues and demonstrate there is a far broader range of safety issues identified in mental health care services than the threat to self and others that underlies the dominant risk‐management approach within mental health services. These issues range from the distal such as under‐resourcing of NHS services, to the proximal such as interaction with the individual practitioner. Previous research reporting safety issues in mental health services has referred to only a small number of concerns, whereas we identified 70 different issues. The majority of those previously reported in a safety context locate the risk within the service user, for example, self‐harm, suicide and violence.^11^ These did feature but were not mentioned with any great frequency by this sample whose responses were dominated by staff factors such as incompetence, negative attitudes and poor listening skills. Locating the risk within the service user leads to a focus on physical safety, often managed through use of seclusion or restraint in inpatient settings, or detention under the Mental Health Act for those in the community. However, locating the threat within individual staff runs the risk of individualising a systemic problem. An under‐resourced system greatly increases the risk of burnout in individual staff, features of which include emotional exhaustion, detachment and poor mental health leading to poor care.^23^ Locating risk within a broader system context allows a broader consideration of safety issues, not limited to physical safety and carries with it the potential to promote positive outcomes.

These data identify that patients feel their safety is at greater risk from service failures such as poor interaction with staff and the impact of short staffing than they are from self‐harm or suicide. This might reflect service users’ perceived lack of influence over the care they receive, of the power imbalance between service providers and those who want or need to use them.^24^ Staff also spoke of poor attitudes and behaviours amongst their number, although this was often in the context of severe staff shortages making it almost impossible to give individual service users the time and attention they need. Significantly, fewer professionals working in inpatient services reported staff attitudes as a safety issue when compared with service user and carer perspectives. This finding is resonant of research comparing service user and staff ratings of the quality of therapeutic relationships where staff rated the quality higher than service users.^25^ This might suggest that professionals have a tendency to be overly optimistic about the nature of their interactions, an issue that might benefit from more reflexive practice or further investigation. Previous research about the triggers of incidents of aggression shows that interactions with staff are a primary cause.^26^ The disparity between service users’ and professionals’ perceptions of attitude as a safety issue may reflect why these encounters frequently trigger incidents. That both service users and professionals with experience of inpatient environments were more likely to report staffing levels as a safety issue implies that this is a particular concern in this setting, which is where the most severely ill service users are likely to be.

The inadequacy of community services as a safety issue was highlighted with service users reporting particular difficulties accessing crisis services and professionals perceiving all community provision as lacking, for example access to CMHTs, specialist teams and day services. There was a clearly perceived threat from the broader climate of reduced public spending that has led to cuts in overall mental health service provision, benefit entitlement and valued community services.

Mapping the responses onto the YCCF clarifies these priorities showing both groups concerns about individual staff factors, staffing levels and the related problems with service process. The amendments made to broaden the scope of the YCFF to include community services and take account of the importance of social as well as physical environment in mental health care provide a framework within which further research and interventions can be developed. One way in which it is feasible to involve patients in safety improvements is by encouraging them to provide feedback on the safety of the care they are receiving. One such tool that uses the YCFF as an underpinning framework is the Patient Measure of Safety now validated for use amongst all general hospital patients.^27^, ^28^ Similar work in mental health care using the YCCF‐MH may provide an opportunity for improving the safety of these services.

### 4.1 Recommendations for practice

This survey identified a wide range of concerns held by service users and professionals but they mostly fell into three categories: individual staff characteristics, service process and staffing levels, and there is a fundamental tension between them. Understaffed services will inevitably struggle to respond to service users’ needs due to constraints on professionals’ time and personal resources. This is not to say that all professionals have skills to deliver care in a compassionate and considerate manner, but that many that do are likely to be hampered by the circumstances in which they are working. That these individual staff characteristics were identified as safety issues is not something that has featured in the literature previously. Not being listened to, believed or feeling able to trust staff can make service users feel unsafe. This suggests these are important aspects of positive relationships and highlights the importance of professionals being able to develop them with service users and carers, but crucially that services are adequately resourced to make this possible. To make improvements to practice, it is recommended that direct collaboration takes place with service users to address the safety needs they have identified.

### 4.2 Limitations

There are limitations to using an opt‐in survey although it was widely publicised using twitter and resulted in a large number of respondents reporting experiences and perspectives. As with all surveys, there was little potential for verifying responses, particularly in a novel area such as this where there are no other data sets that might have been used for comparison. Using twitter as a recruitment mechanism runs the risk of reaching people with the same interests and views, an “echo chamber” effect which has been found to occur with political affiliations.^29^ However, social media users with interests in mental health care are by no means a homogenous group, the advantage of social media being that it provides an equal platform for people to participate in discussion from many different perspectives, including those often deemed “hard to reach” in traditional research sampling. Use of the internet allowed people to participate anonymously, which is of particular relevance to participants who may feel unable to publically state their concerns. The sample was also, of course, limited to those with internet access and the demographics showed that men, black, Asian and minority ethnic (BAME) groups were under‐represented. Additionally, the relatively low number of carer participants prevented separate analysis of this group who might have different needs to service users, as has been reported in research about suicide.^30^We used the term “carer” as we consider this a broad term that can include whoever identifies with this role. However, we appreciate that some family members or friends might not identify as carers despite still having significant involvement and therefore might not have responded to this survey. Further research should be targeted at these specific groups, particularly BAME service users as research has shown they are more likely to experience coercive measures and it may be that their experiences paint a starker picture still.

### 4.3 Future research

Future coproduced research should aim to explore these numerous safety issues in greater depth. Qualitative data would illuminate many of the issues and in turn inform the development of interventions to address systemic safety issues such as optimum staffing levels as well as individual staff factors such as burnout.

## 5 CONCLUSION

This study updates knowledge on patient safety issues in mental health care for the first time in over a decade. It shows that service users, carers and professionals have considerable concerns about the manner in which staff interact with service users, access to support and inadequate staffing levels. Future research should focus on coproducing interventions with service users and carers to improve safety in mental health carer services by focusing on these areas of concern.

## COMPETING INTERESTS

The authors declare they have no competing interests.
